# Sex Differences in Molecular Mechanisms of Cardiovascular Aging

**DOI:** 10.3389/fragi.2021.725884

**Published:** 2021-09-10

**Authors:** Vanessa Dela Justina, Jéssica S. G. Miguez, Fernanda Priviero, Jennifer C. Sullivan, Fernanda R. Giachini, R. Clinton Webb

**Affiliations:** ^1^ Graduate Program in Biological Sciences, Federal University of Goiás, Goiânia, Brazil; ^2^ Araguaia Valley University Center (UNIVAR), Barra do Garças, Brazil; ^3^ Cardiovascular Translational Research Center, University of South Carolina, Columbia, SC, United States; ^4^ Department of Physiology, Medical College of Georgia at Augusta University, Augusta, GA, United States; ^5^ Institute of Biological Sciences and Health, Federal University of Mato Grosso, Barra do Garças, Brazil

**Keywords:** sex differences, aging mechanism, oxidative stress, autphagy, telomerase, RNA, inflammation

## Abstract

Cardiovascular disease (CVD) is still the leading cause of illness and death in the Western world. Cardiovascular aging is a progressive modification occurring in cardiac and vascular morphology and physiology where increased endothelial dysfunction and arterial stiffness are observed, generally accompanied by increased systolic blood pressure and augmented pulse pressure. The effects of biological sex on cardiovascular pathophysiology have long been known. The incidence of hypertension is higher in men, and it increases in postmenopausal women. Premenopausal women are protected from CVD compared with age-matched men and this protective effect is lost with menopause, suggesting that sex-hormones influence blood pressure regulation. In parallel, the heart progressively remodels over the course of life and the pattern of cardiac remodeling also differs between the sexes. Lower autonomic tone, reduced baroreceptor response, and greater vascular function are observed in premenopausal women than men of similar age. However, postmenopausal women have stiffer arteries than their male counterparts. The biological mechanisms responsible for sex-related differences observed in cardiovascular aging are being unraveled over the last several decades. This review focuses on molecular mechanisms underlying the sex-differences of CVD in aging.

## Introduction

Advances in health-assistance, the progress of modern medicine and access to environmental sanitation are some factors favoring the aging population around the world, as observed in the past few decades. According to the World Health Organization (WHO), “elderly” is defined as a chronological age of 60 years old or older (Steverson, 2018). However, chronological age is not a precise marker for the aging process, since biologically, human aging occurs due to cellular damage occurring over the life span. The accumulation of physical, environmental, and social factors regulates a variety of molecular mechanisms enabling aging processes (Davalli et al., 2016). The understanding of the aging process was improved by the Strehler’s enunciation (Strehler, 1977) of the so-called 4 rules of aging. This rule states that aging processes are universal, progressive, intrinsic and deleterious (De La Fuente, 2008), and therefore, will occur in a different pattern among individuals.

The current estimate is that 1 in 11 people in the world is over the age of 65 and by the year 2050, this incidence may increase to 1 in 6 people. Therefore, these numbers bring to the light the necessity of a deeper public discussion regarding older people all over the world.

Aging is a complex degenerative biological process where, over time, the accumulation of multiple irreversible injuries occurs both in molecular and cellular levels, increasing the risk of diseases and eventually, leading to death (Strehler, 1977; De La Fuente, 2008; Bai, 2018). Although aging is not a disease, it can strongly increase the chances for the appearance of degenerative diseases (Bai, 2018).

There are several theories proposed to explain the aging process and they can be divided into two large groups: (Steverson, 2018) genetic programming, and (Davalli et al., 2016) epigenetic changes (Hayflick, 2007). In spite of what is published, there is no conclusive evidence that genes directly drive the changes seen during aging. Therefore, random events (epigenetic) appear to play a major role in the aging process through morphological, physiological and behavioral modifications occurring at all levels of organization (De La Fuente, 2008).

Over the course of life, the functional status of a healthy individual can be predicted by biological parameters (aging’s biomarkers). In fact, they are better predictors for morbidity and mortality compared to chronological age [(CA) – the number of years a person has been alive]. Unlike CA, the biological age [(BA) - how old a person seems to be] is calculated based on genetic, environmental, and disease factors (Bai, 2018). The aging process is complex and thus, aging biomarkers are composed of multiple genes, proteins, and metabolites. The aging biomarkers are divided based on their primary function or physiological target. Alternatively, these biomarkers may have impact on the phenotype and functional evaluation of aging (Bai, 2018).

As age progresses, cells, tissues, and organs start to decline and molecular hallmarks of aging can be seen. Among these, some are related to DNA alterations, including epigenetic changes, genomic instability and telomere loss. Others changes are related to stem cell depletion, , mitochondrial dysfunction, protein loss, malnutrition, intercellular communication changes and oxidative stress (Kuilman et al., 2010; López-Otín et al., 2013). Further, senescence produces proinflammatory and matrix-degrading molecules, in a process which originates in the senescence-associated secretory phenotype [SASP; (distinctive secretome consisting of secretion of many factors, including several cytokines, growth factors, proteases and chemokines)].

Hence, tissue-repair capacity is lost during senescence in progenitor cells, mainly due to mechanisms related to cell cycle disarrangements. However, senescence includes other markers, besides cell cycle disarrangements (Childs et al., 2015; Calcinotto et al., 2019). Of importance, several markers of senescence can be identified, such as higher activity of senescence-associated β-galactosidase (SA-β-gal) (Ravelojaona et al., 2009); augmented levels of cell cycle inhibitors, including cyclin-dependent kinase inhibitor 1 **(**p21^Cip1^) (García-Fernández et al., 2014), cyclin-dependent kinase inhibitor 2A (p16^INK4a^) (Serrano et al., 1997) and cyclin-dependent kinase inhibitor 1B (p27^Kip1^); as well as expression of plasminogen activator inhibitor-1 (PAI-1) (Vaughan et al., 2017), tumor protein (p53) (Serrano et al., 1997) and ARF tumor suppressor (p19^ARF^) (Serrano et al., 1997). Moreover, changes in cellular structures are visible, in addition to accumulation of several subproducts, including: lipofuscin (Georgakopoulou et al., 2013), embryonic chondrocyte-expressed 1 (DEC1), decoy death receptor 2 (DCR2) (Collado et al., 2005), senescence-associated heterochromatin foci formation (Narita et al., 2003), DNA damage foci (Hewitt et al., 2012), senescence-associated distension of satellites (Swanson et al., 2013) and, upregulation of some microRNAs (miRNAs) (Calcinotto et al., 2019).

The risk factors for cardiovascular diseases (CVD) are directly affected by factors related to biological sex and gender differences are related to different rates of mortality among sexes. Yet, the literature supports the idea that biological age and vascular senescence directly impacts sexual dimorphism, and therefore, these factors may represent major mechanisms related to sex differences in the pathogenesis of cardiovascular diseases (Colafella and Denton, 2018).

In this regard, accumulating studies have shown sex-dimorphism in longevity phenotypes. Interestingly, most of the centenarians are women, and female neonates are more likely to survive to childhood (Colafella and Denton, 2018). Indeed, females display increased life expectancy compared to males in several countries (Rochelle et al., 2015). In addition to humans, female laboratory rats live longer than their male counterpart (Berg and Simms, 1960; Viña et al., 2005). Global data for 2015–2020 show that life expectancy for women exceeds 4.8 years of that for men. It is expected that in 2050, the female population aged 65 or over will represent about 54%. The proportion of the female population aged 80 and over will decrease from 61% (2019 data) to 59% in 2050 (United Nations, 2019). Besides all the evidence regarding sex-differences in aging, there is an urgency for more studies directly comparing aging-related mechanisms between sexes (Viña et al., 2005).

In the following paragraphs, some mechanisms involved in cardiovascular aging will be discussed, and evidence relating them to sex-differences will be presented.

## Aging Mechanisms

**FIGURE 1:**
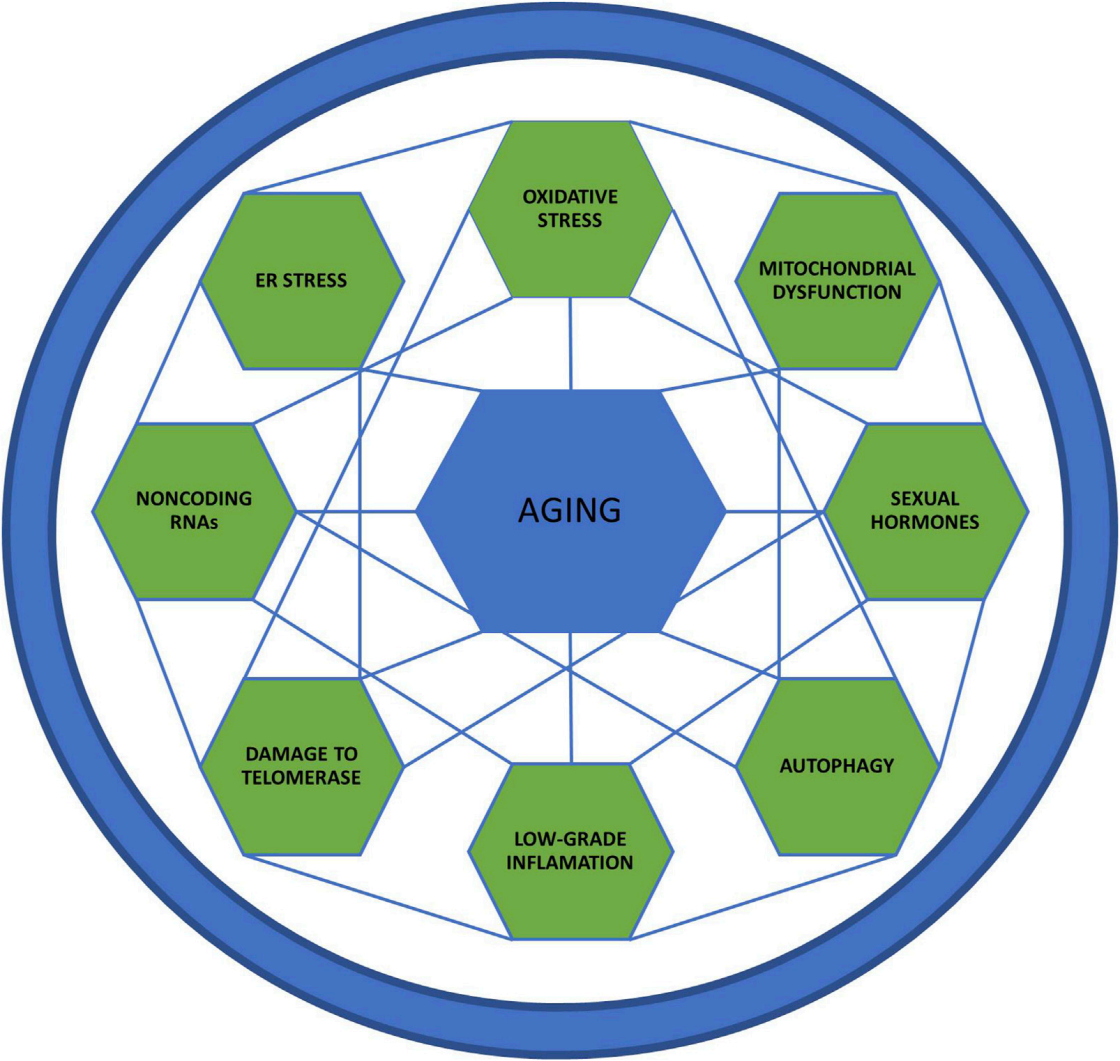
Aging mechanisms. This figure represents the aging mechanisms and the interrelationship between the several intrinsic factors that affect this process.

**FIGURE 2:**
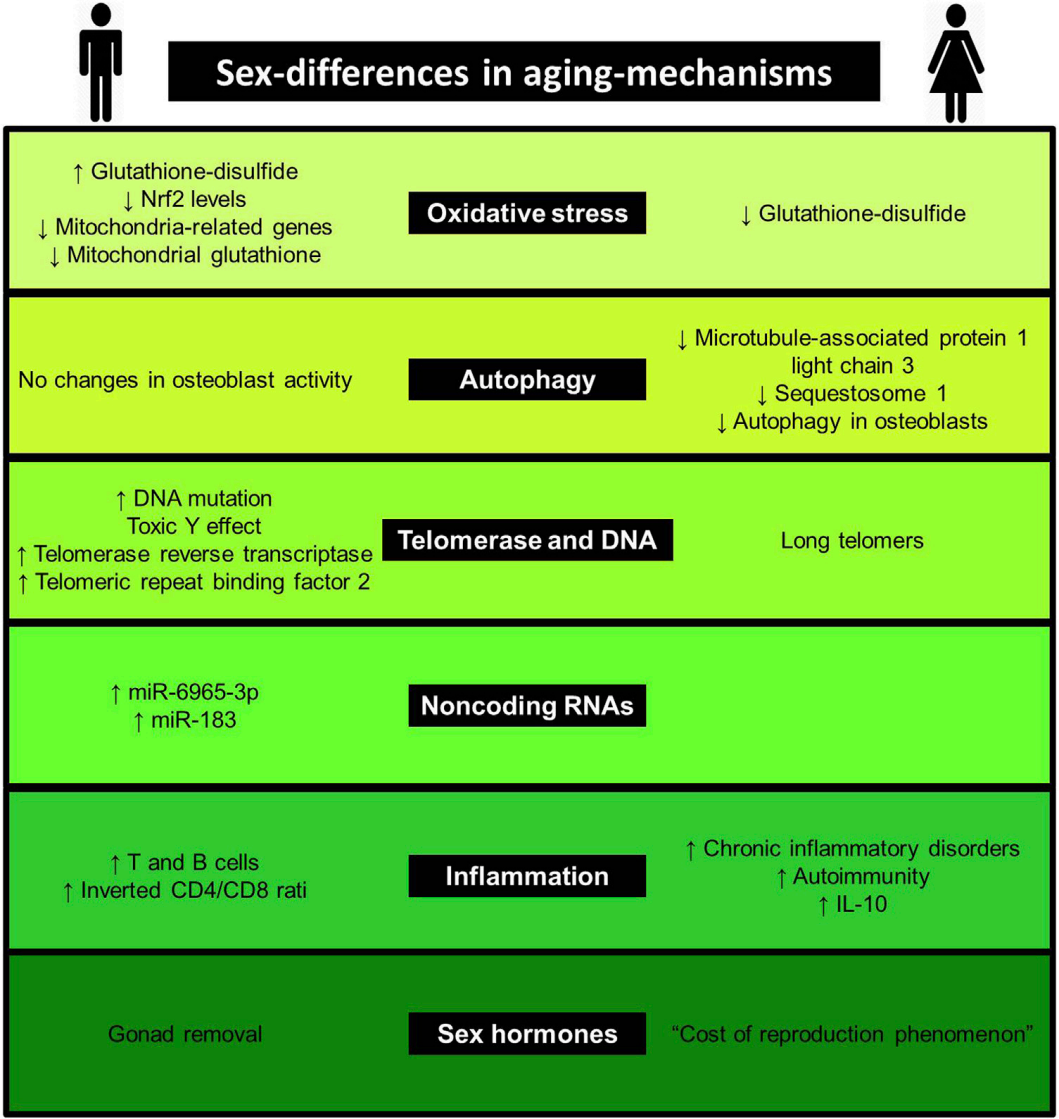
Sex differences in aging mechanisms. This figure shows evidence linking sex-differences to aging-mechanisms.

Aging is a complex process, involving several mechanisms, such as genomic instability, telomere attrition, epigenetic alterations, loss of proteostasis, deregulated nutrient-sensing, mitochondrial dysfunction, cellular senescence, stem cell exhaustion, altered intercellular communication, inflammation, immune aging, oxidative stress, autophagy and noncoding RNAs [for more details, see reviews (López-Otín et al., 2013; Aunan et al., 2016; Dodig et al., 2019; Guerville et al., 2020)]. Considering the complexity of the issue, we limit our review to the most studied mechanisms related to aging, presented in Figure 1. Evidence linking sex-differences in mechanisms are listed in Table 1 and Figure 2 *.*

**TABLE 1:** Evidence linking sex-differences to aging-mechanisms.

Mechanism-related to aging	Specie		Reference
***Oxidative stress- related mechanisms***		
	Increase in glutathione-disulfide (GSSG) in old men whereas aged women displayed a significant decrease expression	Human, muscle biopsy	Pansarasa et al. (2000)
	Decreased Nrf2 levels which resulted in lower expression of detoxifying and antioxidant enzymes in old male mice compared to old female mice	Mice	Pellegrini et al. (2017)
	Mitochondria related genes: Decreased lifespan preferentially in males	Drosophila	Camus et al. (2012)
	Mitochondria related genes: Down-regulation of mitochondrial genes in males, compared to females with similar age	Mouse and human brain	Xu et al. (2007); Berchtold et al., (2008)
		Monkey heart	Yan et al. (2004)
		Human and mouse heart	Isensee et al. (2008)
	Mitochondrial glutathione (GSH) is decreased by 40% in males than females. Ovariectomy decreases mitochondrial GSH levels to values similar to those found in males and estrogen replacement therapy is able to return to the high values	Rats	Viña et al. (2003)
***Increased autophagy***		
	mRNA expression for microtubule-associated protein 1 light chain 3 (LC3) and sequestosome 1 (p62), markers to monitor autophagy, were lower in females than in males	Mice - skeletal muscle and spinal cord tissue	Oliván et al. (2014)
	Osteoblasts and osteocytes from male and female mice decrease with age. However, autophagy in osteoblasts is decreased in old females whereas no changes in this activity is observed in males. Osteoblast- specific autophagy-deficient mice is associated with aging and estrogen deprivation	Mice – osteoblast and osteocyte	Camuzard et al. (2016)
***Damage to telomerase and DNA***		
	Women have longer telomeres than men, indicating an association of longer telomeres with survival improvement	Human	Barrett and Richardson (2011), Gardner et al. (2014), Lapham et al. (2015)
	Age-adjusted mutation DNA load incidence is higher in men than in women as also as somatic mutation accumulation began a decade earlier in male compared to female	Human	Podolskiy et al. (2016)
	A reduction in longevity by 2-years is observed in individuals with Klinefelter syndrome (XXY karyotype), whereas those with an XYY karyotype have a 10-years reduction, suggesting a strong toxic Y effect in humans	Human	Stochholm et al. (2010)
	Centenarian females display balanced XCI expression, which is associated with faster aging and a shorter lifespan	Human	Gentilini et al. (2012), Chuaire-Noack et al. (2014), Ostan et al. (2016)
	Men had significantly greater telomerase reverse transcriptase (hTERT) and telomeric repeat binding factor 2 (TRF2) responses to the acute exercise as compared to women, regardless of age	Human	Cluckey et al. (2017)
***Noncoding RNAs***		
	Age- and sex-related miRNAs expression, indicating thymus atrophy in male mice. Expression levels of miR-6965-3p is higher in male mice than female mice)	Mice - thymus	Guo et al. (2017)
	A single miRNA (miR-183) showed sex-biased expression being expressed at higher levels in males than females	Mice - liver	Kwekel et al. (2017)
***Low-grade inflammation***		
	Aging-related changes to the immune system occurs earlier in males than female	Human	Gubbels Bupp, (2015)
	Decline of T and B cells are greater in men than in women	Human - Japan	Hirokawa et al. (2013)
	Inverted CD4/CD8 ratio was significantly higher in men compared to women	Human - Sweden	Strindhall et al. (2013)
	Increased predisposition to chronic inflammatory disorders and autoimmunity is seen in women, however, compared to men, females exhibit a stronger innate and adaptive immune response	Human	Klein and Flanagan (2016)
	Older women experience increased serum concentration of IL-10, whereas men are characterized by augmented inflammation	Human	Klein and Flanagan (2016)
***Sex hormones***		
	“Cost of reproduction phenomenon”: animals that reproduce are shorter-lived than animals do not	Drosophila, nematode and cricket	Harshman and Zera (2007)
	In many species, including humans, gonadectomy increases longevity of both males and females	Human and dogs	Min et al. (2012), Hoffman et al. (2018)
	The median longevity of neutered men is 14 years longer	Human – United States	Hamilton and Mestler (1969)
	The median longevity of neutered men is 14–19 years longer	Human - Korea	Min et al. (2012)

### Oxidative Stress-Related Mechanisms

Oxidative stress occurs due to the imbalance between overproduction of reactive oxygen species [(ROS), produced by enzymes including NAD(P)H oxidases (Nox), xanthine oxidase (XO), uncoupled nitric oxide synthase (NOS), mitochondrial oxidase, myeloperoxidase (MPO)] and reduced antioxidant mechanisms [superoxide dismutase (SOD), catalase, glutathione peroxidase (GPx)]. The Free Radical Theory of Aging, described in the 1950s by Harman, first described ROS-accumulation linked to biomolecular damage, favoring cell senescence (Harman, 1956), and the concept that ROS accumulation results in vascular dysfunction during aging is well accepted (Genestra, 2007; Liguori et al., 2018). Currently, it is also known that accumulation of reactive nitrogen species (RNS) is also a player in vascular aging (Daiber et al., 2020).

Excessive ROS and RNS elicit oxidation of proteins, DNA and lipids, leading to cellular dysfunction. ROS are also involved in several vascular signaling pathways related to pro-inflammatory cytokine production (El Assar et al., 2013), contractility/relaxation imbalance (Taniyama and Griendling, 2003), calcium homeostasis (Kozai et al., 2014; Liguori et al., 2018), function and morphology, apoptosis (Dimmeler and Zeiher, 2000), senescence and autophagy, among others (Harvey et al., 2015).

Further, in aging, repair systems decline. Of importance, proteasomes, which enzymes are responsible for degradation of damaged proteins, as well as antioxidant enzymes might be modulated by Nrf2/EpRE intracellular signaling. Accumulating data suggest that declining efficiency of Nrf2/EpRE signaling results in age-dependent decline in the antioxidant enzyme (Zhang et al., 2015). In fact, it has been reported that in older rats decreased nuclear Nrf2 protein levels, as well as decreased antioxidant enzymes occurs in several tissues (Shih and Yen, 2007), including liver (Suh et al., 2004), aorta (Ungvari et al., 2011) and carotid arteries. Similar findings have been reported for VSMCs (Ungvari et al., 2011) from monkeys compared to young controls. Moreover, aging phenotypes are observed with the deletion of Nrf2 , favoring hearing loss (Hoshino et al., 2011), skin aging (Hirota et al., 2011) and decreased serum testosterone level (Chen et al., 2015) from Nrf2-knockout mice.

Mitochondrial dysfunction and endoplasmic reticulum stress may also be important sources of ROS, favoring vascular aging. The endoplasmic reticulum (ER) is an organelle, where folding of secreted proteins occurs, synthesis of lipids and sterols takes place and calcium is stored. ER stress occurs when the demand for protein folding and the capacity of the ER for protein folding are misaligned. ROS production elicits prolonged ER stress, favoring apoptosis (Gong et al., 2017). Yet, aging elicits ER stress evoking oxidative damage and endothelial dysfunction (Lenna et al., 2014).

The major function of mitochondria is to provide energy metabolism through the production of adenosine triphosphate (ATP), by oxidative phosphorylation (Payne and Chinnery, 2015). In addition, mitochondria are involved in other cellular processes, including the formation of reactive species (Abate et al., 2020), β-oxidation of fatty acids (Houten et al., 2016), protein metabolism (Hewton et al., 2021), regulation of apoptosis (Abate et al., 2020), maintenance of calcium concentration in the mitochondrial matrix (Finkel et al., 2015), and others. Aging impacts mitochondrial function, favoring damage to mitochondrial DNA (mtDNA). mtDNA differs from nuclear DNA (nDNA) in that it has a circular organization and differentiated coding. Mitochondria are sources of ROS and RNS, which cause damage to mtDNA because unlike nDNA, it is not protected by histones (Gaziev et al., 2014). The mtDNA is susceptible to mutation in the elderly heart, however, despite the mutations, it has been shown not to affect its copy number. In aging, there is an elevation of mitochondrial mutations, since the enzyme 8-oxodeoxyguandine glycosylase responsible for repairing damaged mtDNA is inactivated (Lesnefsky et al., 2016; Hoppel et al., 2017). The increased propensity to stress in the elderly heart has a close relationship with altered mitochondrial metabolism. With aging, the heart has impaired metabolic flexibility, decreasing the ability to oxidize fatty acids and, increasing the dependence on using the glycolytic pathway (Hyyti et al., 2010; Lopaschuk et al., 2010). In addition, there is a decrease in the activity of complexes III and IV responsible for the weakening of breathing. Defective mitochondria increases the production of ROS, oxidative damage and cell death (Müller-Höcker, 1992). The elevation of ROS further exacerbates mitochondrial damage, called the vicious cycle of the mitochondria, by interrupting the cycle of tricarboxylic acid and the electron transport chain (Lu and Finkel, 2008).

### Increased Autophagy

Autophagy is a cellular process of self-degradation with the purpose of maintaining homeostasis, degrading proteins, and renewing organelles (Salabei and Hill, 2015). Cellular aging promotes the accumulation of damaged proteins and organelles that cannot be regulated efficiently by autophagy as this process is impacted by age (Nair and Ren, 2012). Although autophagy is important for cell survival, its excess also promotes cell death (Yu et al., 2006). Therefore, the plasticity of vascular cells should be a well-controlled process.

Evidence shows that aging contributes to a gradual decrease in autophagy and activity of the ubiquitin protein system, resulting in functional deterioration of the organism (Ma et al., 2011). Autophagy is initiated by Beclin-1 and phosphatidylinositol 3-kinase class III (PI-3K). A complex between Beclin-1 and PI3K-III plays an important function in membrane traffic and restructuring involved in autophagy, phagocytosis and endocytosis (Zhong et al., 2009). The autophagy genes ATG5, ATG7 and BECN1 are downregulated in the brain of older people when compared to young people (Lipinski et al., 2010). In cardiomyocytes, the main autophagic signaling pathways are MLC/FAK/AKT/mTOR-mediated inhibitory pathways and Beclin 1 activation pathways (Fernández et al., 2018).

Due to aging, there is a decrease in cardiac autophagy, resulting in loss of homeostasis in cardiac tissue and consequent dysfunction (Taneike et al., 2010). The regulation of autophagy occurs positively by AMP-dependent protein kinase (AMPK) and, conversely, Akt and mTOR downregulate AMPK through the phosphorylation of their targets as p70s6k (Sarkar et al., 2009). Of importance, the interruption of mTOR is the main factor for the reduction of longevity (Blagosklonny, 2008). Moreover, it has been demonstrated that rapamycin, a specific inhibitor of mTOR, was able to increase lifespan in several species (McNab, 2003; Kapahi et al., 2004; Powers et al., 2006; Harrison et al., 2009) due to its ability to suppress cell growth and proliferation (Gao et al., 2020). In humans, inhibition of mTOR pathway improves age-related disorders such as heart disease (McCormick et al., 2011).

Thus, genetic modifications are alternatives to increase autophagy and favor cardiac function, the removal of dysfunctional organelles and poorly folded proteins (Taneike et al., 2010). Akt/mTORC1 inhibition, related to increased autophagy and; increased HSP27 expression, related to reduced accumulation of LC3-II and p62; are mechanisms linked to the attenuation of damage related to cardiac aging. These changes related to autophagy are able to improve contraction, reduce oxidative stress and resist cardiomyocyte senescence (Lin et al., 2016; Gao et al., 2020). In addition, microRNAs such as miR-497-3p, miR-26b-5p or miR-204-5p when negatively regulated increase myocardial autophagy (Qi et al., 2020).

### Damage to Telomerase

Telomeres are non-coding, repetitive sequences of nucleotides, physically localized in the terminal part of chromosomes and associated specialized proteins that interact with DNA to form nucleoproteins (De Lange et al., 2006). Aging is mainly determined by the length of telomeres and cells have a limited number of divisions. After each division the telomere is shorter until it reaches an indivisible state known as replicative senescence (Chan and Blackburn, 2004; Martin, 2011).

Among the processes related to telomere shortening are stress, chemical damage, nuclease action or age progression. The activity of telomerase, a specialized RNA-dependent polymerase, compensates for this loss in a mechanism that replicates the ends of linear DNA molecules. The telomerase RNA (TERC) and telomerase reverse transcriptase (TERT) forms a telomerase complex (Lane and Martin, 2010; Scarabino et al., 2019).

When investigating the relationships of TERC and TERT genetic variations associated with aging, it was observed that decreased life expectancy may be related to genotypes including TERT VNTR MNS16A L/L and TERTOs rs2853691 A/G or G/G (Scarabino et al., 2019). In addition to telomerase activity, another factor that interferes with the shortening of the telomere is the loss of protection and stability due to irreparable damage to DNA. This occurs by destabilizing Shelterin complex components such as telomeric factor 1 and 2 (TRF1 and TRF2) (Martínez et al., 2009; Ryan et al., 2010; Porro et al., 2014).

In unprotected telomeres, structure regulation and processing by lncRNA TERRA (RNA containing telomeric repetition) transcribed from telomeres occurs (Porro et al., 2014). The elevation of TERRA expression may be linked to the senescence process. After DNA damage, there is an increase in TERRA signaling by RNA polymerase II, which is linked to the chromatin structure. In damaged telomeres, a p53-dependent increase in the level of EARTH occurs (Caslini et al., 2009; Porro et al., 2010; Porro et al., 2014).

In some cases, as in human peritoneal mesothelial cells (HPMCs), telomerase is active, however, they have short telomeres (3.5 kbps). Reduced telomerase activity plus accompanying mitochondrial dysfunction, resulting in ROS production may explain this phenomenon. Thus, oxidative stress contributes deeply to disruption of non-telomeric regions, promoting premature aging (Ksiazek et al., 2007; Passos et al., 2007).

Excessive ROS production favors the migration of hTERT from the nucleus to the cytosol, reducing telomerase activity and consequently promoting aging of endothelial cells. The use of antioxidants and other active ingredients can delay this process. N-acetylcysteine incubation decreased ROS formation, preventing damage to mtDNA. In addition, incubation with atorvastatin had similar effects (Haendeler et al., 2004; Santos et al., 2004; de Punder et al., 2019). It is important to note that despite the beneficial effects of antioxidants against ROS in several animal models and *in vitro* studies, many large-scale randomized controlled trials have provided inconsistent results on the prevention of chronic diseases and lifespan in humans (Sadowska-Bartosz and Bartosz, 2014).

Metabolic stress affects the size of the telomeres. For example, a decrease in serum omega-3 levels has a correlation with telomere size, which can be a marker of aging (Farzaneh-Far et al., 2010).

Evidence from humans studies show that elevated ROS contributes to chronic inflammatory diseases and shortening of telomeres (Finley et al., 2006; Letsolo et al., 2017). Acceleration in the telomere shortening is drastically observed in oxidative stress conditions by several mechanisms: 1) single strand breaks (SSB) mediating the collapse of replication fork and the loss of telomere; 2) increased cell division and telomere shortening due cell death and/or senescence-related process, 3) multi-telomeric foci at chromatid ends termed fragile telomeres, due to unreplicated ssDNA accumulation (Von Zglinicki, 2002; Sfeir et al., 2009).

### Noncoding RNAs

Epigenetic alterations act as a link between the intrinsic genetic landscape and extrinsic environmental influences, including DNA methylation or hydroxymethylation, histone modifications, and chromatin remodeling including microRNAs (miRNAs), and noncoding RNAs (ncRNAs) (Greco and Condorelli, 2015; Venkatesh and Workman, 2015; Zhang et al., 2018). Noncoding RNAs are a class of regulatory ncRNAs and can provide important data for the aging process. They have a transcription length greater than 200 nucleotides and can be separated according to their length into short and long ncRNAs (Jin et al., 2019).

Circular RNA (circRNA), a recently reported type of noncoding RNA, has been shown to play a role in both cellular senescence and cellular survival, and some recent studies make the assumption that circRNA contributes to the pathogeneses of age-related diseases, making them possible biomarkers (Cai et al., 2019). Age-related diseases are related to processes of proliferation and cell survival, which are also controlled by ncRNAs, modulating some pathways via cyclin-dependent kinase inhibitor 1 (p21), cyclin-dependent kinase 2 (CDK2) and mammalian forkhead transcription factors (Maiese, 2016).

Studies have profiled the changes in lncRNAs in CVD and aging. Cardiac-specific lncRNAs from mouse hearts with myocardial infarction have been associated with maladaptive cardiac pathological remodeling and some of these lncRNAs (CDKN2BAS1/ANRIL, RMRP, RNY5, SOX2-OT, SRA1 EGOT, H19, HOTAIR, and LOC285194/TUSC7) are changed (2,204 genes are up-regulated and 1,338 genes are down-regulated) in individuals with dilated cardiomyopathy and aortic stenosis [116]. Moreover, expression of the myosin heavy-chain-associated RNA transcripts (Myheart or Mhrt, a conserved lncRNAs involved in the antisense direction) increases as age progresses and acts as a safeguard for cardiac health in mice and humans (Greco et al., 2016).

Besides lncRNAs, circRNAs have been linked to aging-associated genes, such as circANRIL. In fact, circRNAs lead to greater proliferation of SMCs during atherosclerosis, and they are increased in patients with coronary artery diseases (Holdt et al., 2016; Ghafouri-Fard et al., 2021).

Another gene involved in decreasing longevity is the LMNA that encodes laminin A and the detection of its mutation can be used to track damage by age (Zhavoronkov et al., 2012). Mutations in genes that encode receptors, kinases, substrates of metabolic pathways of insulin/IGF-1, PI3K, TOR, MAPK can also occur, resulting in an increase or decrease in life span (Clancy et al., 2001; Narasimhan et al., 2009; Zhavoronkov et al., 2012).

In patients with myocardial infarction, the lncRNAs called aHIF, KCNQ1OT1, MALAT1 are more expressed while those of ANRIL are decreased when compared to healthy people. In mice with the same condition, MIRT1 and MIRT2 are stimulated and correlated with genes involved in left ventricular remodeling and ejection fraction (Vausort et al., 2014; Zangrando et al., 2014).

In heart failure, common in aging, there is positive regulation of some lncRNAs such as NRON (NFAT non-coding repressor) and MHRT (RNA transcripts associated with the myosin heavy chain) (Vausort et al., 2014) and mitochondrial LIPCAR (uc022bqs.1) with chimeric fusion transcription of the 5′ end of COX2 and 3′ end of CYTB (Kumarswamy et al., 2014). The MHRT-Brg1/BAF complex can protect the heart from hypertrophy and insufficiency (Han et al., 2014).

In hypertensive patients there is an increase in plasma levels of lncRNA-AK098656 with VSMCs-dominant specific for humans. This lncRNA binds myosin heavy chain proteins and fibronectin-1, facilitating their degradation. AK098656 may be an efficient therapeutic target for hypertensive control (Jin et al., 2018).

### Low-Grade Inflammation

Aging is also characterized by a chronic low-grade inflammation state, also known as “*inflammaging”*, shifting to the production of pro-inflammatory cytokines (Rea et al., 2018), chemokines, and adhesion molecules (De Martinis et al., 2005; Vasto et al., 2007; Calder et al., 2017). The immunological decline increases the susceptibility to various ailments such as cardiovascular diseases, cancer and, infections which are higher in older people (Weyand and Goronzy, 2016).

With age, the body starts to maintain a chronic pro-inflammatory state, as it is constantly in a stressful environment. Among the inflammatory predictors of aging are an increase in CD8^+^ T cells, decreased CD4^+^ T cells and CD19^+^ B cells, and mitogen-induced inhibition of T cell proliferation. High levels of serum interleukin -6 (IL-6) is a reliable marker of disability and mortality in the older people. Other cytokines such as interleukin-10 (IL-10, anti-inflammatory) and tumor necrosis factor alfa (TNF-α, inflammatory) can also be considered serum markers of aging (Butcher and Lord, 2004; De Martinis et al., 2005). Although necessary, when there is an excess of inflammatory responses, a stimulus to human aging occurs (Xia et al., 2017). In an experiment with older horses, there was an increase in the expression of interleukin-1 beta (IL-1 β), interleukin-15 (IL-15), interleukin-18 (IL-18) and TNF-α in peripheral blood (Xia et al., 2017).

The general balance of cytokines is important in aging, as well as alterations in the gene-promoting regions of these cytokines, thus determining susceptibility to age-related diseases (Lio et al., 2003). A study showed that Italian male centenarians display a more frequent single nucleotide polymorphism (SNP) in the IL-6 gene promoter region. In addition, they also have an increased occurrence of SNP -1082G in the IL-10 gene 5′ flanking region. Therefore, polymorphisms of inflammatory cytokine genes can regulate immunoinflammatory responses (Pes et al., 2004).

A low-grade state of inflammation is observed in aging, further affecting apoptosis and autophagy (Calder et al., 2017). Impaired autophagy can trigger the appearance of a pro-inflammatory phenotype and activation of the inflammasome (Calder et al., 2017), a multiprotein complex that is activated in response to microbial invasion or damage‐associated molecular patterns (DAMPs) in innate immune cells (Guo et al., 2015).

Aberrant activation of NLR family pyrin domain containing 3 protein (NLRP3) inflammasome leads to the production of inflammatory cytokines IL-1β and IL-18 and contributes to amplification of pathological inflammation. Of importance, inflammasome is activated during aging and aging-related disease (He et al., 2020). In fact, ablation of NLRP3-inflammasome protected mice from age-associated changes in the heart. Moreover, old NLRP3 KO mice showed an inhibition of the PI3K/AKT/mTOR pathway and improvement in autophagy, compared with old wild-type mice, suggesting that NLRP3-inflammasome suppression improves longevity and prevents cardiac aging in male mice (Marín-Aguilar et al., 2020).

## Sex Differences in Cardiovascular Aging

During aging, cardiovascular structure and function start a progressive decline. Heart and vasculature gradually show homeostatic imbalance, vascular stiffening, fibrosis, and increased left ventricular (LV) wall thickness leading to accentuated tissue adaptations and decreased stress tolerance (Stern et al., 2003). All the aging mechanisms previously described in this review have been shown to participate in cardiovascular aging.

It is well known that men display increased adverse cardiovascular events compared to pre-menopausal women. However, this statistic inverts after menopause, where this female cardiovascular protection is lost (Pollow et al., 2019; Virani et al., 2020). Moreover, the development of CVD coincides with the decline of female sex hormones such as estrogens (Zhao et al., 2018).

Interestingly, decreased plasma levels of testosterone have been linked with age-related CVD pathways in men (Traish et al., 2009), indicating that abnormal levels of testosterone may have deleterious effects in the cardiovascular system (Lopes et al., 2012). Further, lower testosterone levels, when observed in elderly men, are related to an increased occurrence of heart diseases and modification in body composition. However, hypogonadism is related to augmented cardiovascular risk (Guay, 2009). Regardles, mechanisms involved in cardiometabolic maintenance are clearly regulated by male sex steroids, demonstrating an important role during aging (Traish et al., 2009; Barrientos et al., 2020).

Conversely, men with reduced testosterone levels displayed decreased death risk (Laughlin et al., 2008). And, supraphysiological levels of testosterone may contribute to CVD risk (Gagliano-Jucá and Basaria, 2019). Testosterone impacts vascular function, by overexpression of pro-inflammatory cytokines, arterial thickness and by reducing NO synthesis and bioavailability (Lopes et al., 2012). Mesenteric arteries from old Wistar rats, treated with testosterone displayed augmented oxidative stress and inflammation possibly to due increased leucocyte migration, favoring increased cardiovascular risk (Chignalia et al., 2015). Further, apoptosis and migration of VSMCS are processes induced by testosterone, via ROS formation and MAPK activation, respectively (Lopes et al., 2014).

A prospective cohort of post-menopausal women in the Multi-Ethnic Study of Atherosclerosis showed an association between higher total testosterone/estradiol ratio and increased risk on incident CVD, as well as total testosterone and bioavailable testosterone levels (Wang et al., 2012; Zhao et al., 2018).

The loss of renal function with age or disease can drive an increase in blood pressure (Hall, 2003). A study conducted with hypertensive and normotensive postmenopausal women reported a decrease in sex hormones and increase in sensitivity to sodium intake (Tominaga et al., 1991). Of note, evidence shows increased sodium/hydrogen exchanger 3 (NHE3) expression in the proximal portion of the renal tubule in males compared to females, whereas epithelial sodium channel (ENaC) and sodium/chloride co-transporter (NCC) are higher in distal portions of the renal tubule from females, compared to male rats, favoring sodium excretion in females (Veiras et al., 2017).

Obesity is a risk factor for CVD and has been linked to telomere shortening (Batsis et al., 2018). Interestingly, telomere shortening can induce permanent cellular senescence (Colafella and Denton, 2018), but this process occurs earlier in men, even though telomere length is similar between sexes in later life (Barrett and Richardson, 2011). Moreover, kidneys from male and female rats displayed age-related telomere shortening, however, males were more affected than females. Further, p53 and p21 (senescent markers) expression significantly increased in males, but not in females whereas augmented antioxidants mechanisms (such as SOD, glutathione peroxidase and glutathione reductase) were observed in the cortex from older female (Tarry-Adkins et al., 2006).

CVD-treatment, targeting peculiarities between the sexes, may be an important topic for further investigations. Many medications are commonly used to prevent CVD before it occurs, as well as to prevent existing disease from getting worse. In this regard, treatments for cardiovascular disease are more common in women than men in primary prevention, but the reverse is seen in secondary prevention (Walli-Attaei et al., 2020). Interestingly, a meta-analysis showed that women were significantly less likely to be prescribed with aspirin, statins, and angiotensin-converting enzyme inhibitors, compared to men (Zhao et al., 2020). Therefore, it is fair to say that guideline-recommended medications to treat CVDs are less frequently prescribed to women. Another important point is that women may not be receiving the same degree of health assistance compared to men (Okunrintemi et al., 2018), as revealed by a survey conducted with more than 11 million women, using data from the Medical Expenditure Panel Survey, from the Department of Health and Human Service. The authors also speculate that physicians may have a dubious view of similar symptoms when reported by men, compared to women”

It is reasonable to state that the CVD’s treatment does not often consider the peculiarities inherent to the binomial sex and age. Aging brings a decline in basal physiological functions and in some cases, the patients are as vulnerable to the pharmacological treatments as they are to the CVD (Stuck et al., 2015). Strategies to improve the identification of avoidable risk factors in an aging population, including the prediction of adverse drug reactions, will bring some light to these patients receiving a safer treatment.

In this regard a large gap for the evaluation and selection of safer treatments is still an open question, and personalized medicine is becoming a strong market niche. Personalized medicine goes for the selection of therapies offering the highest safety, ensuring patient care (Vogenberg et al., 2010). On the other hand, making it suitable for a large portion of the population is still a challenge, considering that most CVD’s protocols still work with the concept that “one size fits all” (Riondino et al., 2019).

### Age-Sex Implications in Cardiac Structure

Cardiac remodeling is a process continuously influenced by age and cardiac hypertrophy is common in both sexes (Kane and Howlett, 2018). Over the lifetime, left ventricular (LV) mass and volume are reduced in both sexes. However, structural evaluations suggest bigger LV mass and wall thickness in men, compared to women (Salton et al., 2002; Lieb et al., 2009). Of importance, in women, changes observed in the wall thickness are more accelerated, especially in the face of hypertensive conditions and diabetes (Cheng et al., 2010). Even while receiving anti-hypertensive-drugs, women have higher risk to develop LV hypertrophy, compared to men (Izzo et al., 2017). In fact, the format of remodeling that occurs in women present a more concentric remodeling over the years (Cheng et al., 2010; Merz and Cheng, 2016; Singh et al., 2019; Miller et al., 2020) and this tendency favors diastolic dysfunction and a higher incidence of heart failure with preserved ejection function in women (Gori et al., 2014).

### Extracellular Matrix Deposition

Extracellular matrix deposition is a hallmark for cardiac remodeling in aging and sex differences are also observed. A study conducted in non-diseased human hearts showed an age-dependent sex-specific regulation of extracellular matrix components (Dworatzek et al., 2016). Young women have lower collagen type I, III and VI, tissue inhibitor of metalloproteinase 3 than men. Interestingly, the expression of these proteins was higher in older women, compared to men. In fact, differences in the expression of female cardiac extracellular matrix genes, with excess collagen and other proteins in the LV, and estrogen deficiency in menopause (Dworatzek et al., 2016; Merz and Cheng, 2016). Indeed, cardiac extracellular matrix proteins, including collagen, are greater in the LV of older women than in older men, suggesting a role on heart fibrosis (Dworatzek et al., 2016).

### Estrogen

Estrogen seems to regulate extracellular matrix deposition, suggesting a distinct molecular mechanism occurring between the sexes. *In vitro* experiments conducted in primary human aortic smooth muscle cells exposed to estrogen resulted in decreased collagen, and increased elastin (Natoli et al., 2005). In postmenopausal women, estrogen contributed to decreased LV mass (Lim et al., 1999) and elicited increased baroreflex sensitivity (Huikuri et al., 1996). Yet, estrogen regulates cardiac modulators related to hypertrophy, including natriuretic peptide (Van Eickels et al., 2001; Babiker et al., 2004; Pedram et al., 2005; Pedram et al., 2008), endothelin (Pedram et al., 2005) and, PI3K/PKB-signaling (Camper-Kirby et al., 2001; Bhuiyan et al., 2007). Of importance, LV mass and wall thickness are related to polymorphisms occurring in the *ESR2* gene in women, but not in men (Peter et al., 2005).

### Other Mechanisms

Other possible links to cardiac remodeling are the reduced number of ventricular myocytes, usually observed both in humans and animals, probably caused by several mechanisms. First, decreased stem cell regeneration is observed in men, whereas cardiac stem cells are more expressed in women (Olivetti et al., 1995). Additionally, apoptosis is an event more frequently observed in men facing cardiovascular diseases, compared to women (Boddaert et al., 2005). Yet, other processes such as cardiomyocyte necrosis and autophagy, hypertrophy and fibroblast proliferation are more prominent in men than in women (Zhang et al., 2007; Leon and Gustafsson, 2016; Kane and Howlett, 2018).

The robust body of evidence implicating age- and sex-related cardiac remodeling makes this is an exciting area for future exploration, aiming to define other mechanisms that might be implicated in these events.

### Age-Sex Implications in the Cardiac Function

#### Electrical Activity

A decreased number of cells in the pacemaker has been established during aging, and this is related to a decline in gene expression involved in the sinoatrial function, such as changes in many ion channels and ion-homeostasis related genes (Tellez et al., 2011; Mirza et al., 2012; Kane and Howlett, 2018). The heart decreases its electrical conductance with older age and a slower conduction is seen through the atrium and atrioventricular node. Thus, the occurrence of bradycardia and the necessity of an artificial pacemaker system increases, especially in men (Méndez-Bailón et al., 2017; Chamandi et al., 2018).

One plausible explanation for reduced conduction may be the reduced expression of connexins, involved in myocyte cell connections, in addition to electrophysiological changes in the atrial myocytes (Bonda et al., 2016; Jansen et al., 2017). Experiments using ischemia/reperfusion in hearts isolated from mice, demonstrated that expression of mitochondrial connexin-43 has a protective cardiac effect, and that post-ischemic estrogen treatment reduced infarct size while increasing connexin-43 expression in mitochondria. Further, mitochondrial Cx43 displayed a protective role in female hearts leading to a decreased myocardial ischemia/reperfusion compared to that in age-matched males (Wang et al., 2020). Pacemaker nucleus records, obtained in the electric fish *Apteronotus leptorhynchus*, showed a sex-dimorphism under the influence of steroid hormones (Zupanc, 2020). Pronounced sex-differences in morphology and in the formation of a syncytium by the astrocytes were observed. A larger syncytium area covering the pacemaker cells was present in females, along with greater connexin-43 expression, suggesting a stronger gap-junction in females, compared to males.

#### Systolic and Diastolic Function

Implications coming from sex-related differences in the heart structure also impact cardiac function, such as systolic function, which clinically, may be frequently accessed by the left ventricular ejection fraction (LVEF), a value that is generally higher in women, compared to men (Chung et al., 2006; Tadic et al., 2019). Older women present higher ejection fraction than men, along with higher right ventricular fractional area and a negative global longitudinal strain, besides greater tricuspid regurgitation velocities. Moreover, abnormal ventricular end-diastolic and end-systolic volumes was higher in women than men. However, overall women had better survival outcomes with pulse pressure as a key determinant and, conversely, heart rate and B-type natriuretic peptide were associated with poorer outcome in men (Beale et al., 2019).

Despite some evidence showing increased ejection fraction in aged women, other studies have shown preserved ejection fraction and, therefore, a better knowledge of cardiac structure and function abnormalities during heart failure may help diagnose people with high risk for death due to CVD (Shah et al., 2014; Merrill et al., 2019). In fact, among old patients (49% were women) with preserved ejection fraction, LV hypertrophy and higher pulmonary artery pressure and LV filling pressure were predictive of heart failure, hospitalization and cardiovascular death (Shah et al., 2014).

Cardiac function also differs and men have defects in the systolic pump earlier, while diastolic function is little compromised (Gebhard et al., 2013). On the other hand, women have greater diastolic involvement, greater systolic torsion and LV shortening, in addition to increased ejection fraction (Yoneyama et al., 2012; Gebhard et al., 2013). In older age, the systolic function is reduced, also evidenced by the decreased capacity of the ventricular myocytes to contract (Fannin et al., 2014; Kane and Howlett, 2018).

Diastolic dysfunction has shown to be a hallmark pathological intermediate in the development of HFpEF. In fact, a cohort study in ethnic Asians demonstrated that prevalence of diastolic dysfunction as also others diastolic parameters increase with advanced age and it is greater in women overall than men (Chang et al., 2021).

The maintenance of optimal output involves development of greater systolic stiffness in the LV (Chen et al., 1998). Interestingly, end-systolic elastance is higher, mainly, in older females (Saba et al., 1999; Redfield et al., 2005). The mechanism behind this event may be related to alterations in chamber geometry. Of importance, aging is associated with impaired subendocardial function (Lumens et al., 2006) due to reduced global longitudinal shortening (Støylen et al., 2020) where arterial stiffness is a greater contributor, especially in women (Bell et al., 2017; Yoshida et al., 2020).

Further, female obesity results in heart failure with preserved ejection fraction (HFpEF) (Kitzman and Shah, 2016). This condition occurs due to ischemic events in the heart’s circulation, especially in vessels of large caliber, resulting in reduced contraction of the ventricles (Murphy et al., 2020). This and other evidence show that the occurrence of CVD in obese patients is most dangerous for women. Some mechanisms may explain the association between increased adiposity and HFpEF. Augmented adiposity promotes inflammation which induces dysregulation of the nitric oxide-cyclic guanosine monophosphate-protein kinase G signaling cascade which in turn leads to mitochondrial disruption and endothelial dysfunction (Paulus and Tschöpe, 2013). Additionally, greater activation of the angiotensin-aldosterone signaling pathway can cause myocardial injury (Kitzman and Shah, 2016). Moreover, estrogenic vasodilatory effects as well as myocardial energy substrates changes during post-menopause and they have been proposed as important contributors to the pathogenesis of HFpEF, once its prevalence increases in postmenopausal women (Sabbatini and Kararigas, 2020; Gökçe et al., 2003; Voutilainen et al., 1993).

In fact, hormone therapy (HT) decreased LV mass by 20% in postmenopausal women (Lim et al., 1999) and also reduced LV mass index in hypertensive women (Light et al., 2001). In the same way, ovariectomized animals treated with GPR30 (estrogen receptor) agonist (G1) displayed improved LV diastolic function, collagen deposition, atrial natriuretic factor, cardiac NAD(P)H oxidase 4 (NOX4) expression and, inhibited angiotensin II-induced hypertrophy in H9c2 cardiomyocytes, whereas GPR30 antagonist inhibited the protective effects on this hypertrophy (Wang et al., 2012).

#### Estradiol and Estrogen

Isolated hearts from both adult female and male rats treated with estradiol leads to the development of cardioprotection against infarction, though a reduced effect was observed in hearts from male rats (Sovershaev et al., 2006). The mechanism behind this protection relies on the activation of the PI3K/GSK3β, favoring mitochondrial protection. Further, estradiol is able to induce S-nitrosylated proteins and greater NO-signaling activation, which are known to be cardioprotective (Deschamps and Murphy, 2009). Estrogen deficiency via ovariectomy (OVX) in hypertensive female rats increases oxidative stress as well as cardiac inflammation which result in diastolic dysfunction and myocardial fibrosis (Mori et al., 2011).

Further, estradiol protects isolated hearts from female SHR against IR injury via GPR30 leading to Nocth-1 activation. Through non-nuclear estrogen receptors, phosphoinositol 3 kinase-dependent and mitochondrial adenosine triphosphate (ATP)-sensitive potassium channels survival (Rocca et al., 2018) leads to endothelial nitric oxide synthase (e-NOS) activation and S-nitrosylation (Shao et al., 2016) which play a key role in post-translational modification in cardioprotection (Menazza et al., 2017).

Although several studies showing a cardio-protective role for premenopausal hormones, conflicting evidence exists regarding the efficacy of hormone therapy in postmenopausal women (Korzick and Lancaster, 2013).

Increased collagen deposition and LV stiffness as well as concentric remodeling are associated with female sex and age (Hoshida et al., 2016). Among the female hormones, estrogen plays a key role during age-related diastolic dysfunction in women. Indeed, this hormone is a vasodilator (Reis et al., 1994) acting at cellular Ca^2+^ handling sites, which could impact diastolic performance (Oneglia et al., 2020). Further, it was demonstrated in rats that OVX decreased expression of phosphorylated phospholamban [PLB (facilitates sarco(endo)plasmic reticulum Ca^2+^-ATPase 2a (SERCA2a) activity)] which in turns reduced lusitropy and increased cardiac filling pressures. Of note, the worst diastolic dysfunction caused by OVX was demonstrated in older rats than middle-age (Alencar et al., 2017). Furthermore, estrogen treatment in a primate model of menopause preserved diastolic function due calcium homeostasis (Michalson et al., 2018).

#### Oxidative Stress

Another mechanism contributing to diastolic dysfunction is increased oxidative stress (Kumar et al., 2019). Decreased ROS production was seen in female rat heart upon ischemia/reperfusion compared with age-matched male hearts via posttranslational modification of mitochondrial proteins (Lagranha et al., 2010). Indeed, preclinical ischemia/reperfusion studies with OVX demonstrated that estrogen induces ATP production and electron transport chain activity (Lancaster et al., 2012), downregulates mitochondrial apoptotic pathways (Fliegner et al., 2010) and upregulates mitochondrial antioxidants (Liu et al., 2014). Further, this hormone was able to reduce ROS formation, prevent energy dysregulation and improve diastolic function (Chen et al., 2015) in treated OVX mouse model of hypertrophic cardiomyopathy. Of importance, ROS acts as a scavenger to nitric oxide, a key regulator of normal diastolic function (Silberman et al., 2010).

#### Gonadectomy

As already mentioned, circulating estrogen and testosterone levels decrease with age and there is a link between sex hormones and CVD. Interestingly, long-term gonadectomy in older male mice slowed isovolumic relaxation time promoting diastolic dysfunction in the aging heart. The mechanism behind this dysfunction may due to higher cardiac expression of PLB protein in older mice with gonadectomy compared to control mice. The increase in PLB interferes with Ca^2+^ uptake, prolonging its uptake in the sarcoplasmic reticulum and prolonged transient Ca^2+^ decay in cardiac cells from aging gonadectomy mice. Further, in hearts from aging gonadectomy mice, there is a reduction in phosphorylation of the regulatory myosin light chain ELC (Ayaz et al., 2019).

#### Other Mechanisms

Growing evidence has linked polyunsaturated fatty acids (PUFAs) and cardiac homeostasis. PUFAs are metabolized through numerous metabolic pathways, including cytochrome P450 (CYP) monooxygenase (Jamieson et al., 2017). The progression of CVDs such as hypertension and atherosclerosis has been implicated in the hydrolysis and inactivation of epoxy metabolites by soluble epoxide hydrolase (sEH) (Harris and Hammock, 2013). When compared to aged wild-type (WT) mice, male sEH null mice had preserved diastolic function and females had preserved systolic function. Furthermore, the latter preserved Sirt-3 activity, mitochondrial ultrastructure and SOD activity levels. Increased age-related carbonyl levels have been demonstrated in male WT and sEH null mice (Jamieson et al., 2020).

### Age-Sex Implications in the Cardiac Vasculature

#### Cardiac Vasculature Thickening

In human, macroscopic changes such as thickening of the aortic valve leaflets, accumulation of lipids and calcification of the aortic valve are observed in both sexes during aging. Carotid intima-media thickening, coronary artery calcification and the formation of atherosclerotic plaques are more prevalent in men than in adult and middle-aged women; however, as they become older, women start to present greater dysfunctions (Kelley et al., 2011; Merz and Cheng, 2016). Men tend to have a higher risk of plaque (Ota et al., 2010; Merz and Cheng, 2016). Interestingly, women with coronary artery disease were less likely to undergo optimal secondary prevention with antiplatelet and lipid-lowering therapies than their male counterparts (Madan et al., 2020).

When considering the scientific evidence on the increased risk of CVD in old age, it is important to consider changes in cardiac functional and structural aspects (Kane and Howlett, 2018). Mortality in the western world is mainly caused by CVDs, a factor that is increasing globally (Heidenreich et al., 2011). In the formation and expansion of plaque in atherosclerosis, senescence inducers, such as telomere shortening and oxidative stress are produced in vascular smooth muscle cells and endothelial cells (Wang and Bennett, 2012). As a result, senescent endothelial cells are prone to apoptosis, endothelial layer “leakiness”, oxidized LDL extravasation and, decreased NO secretion (Hogg and Kalyanaraman, 1999; Zhang et al., 2002; Krouwer et al., 2012). Further, with aging, the vasculature displays alterations such as aortic stiffening and thus, vascular aging may be considered a prodromal stage of atherosclerotic diseases (Zhang et al., 2018).

#### Estradiol

The incidence of HFpEF and HFrEF in both sexes, increases with age. In women, especially postmenopausal women, there is a higher HFpEF index, while men have a higher prevalence of HFrEF (Beale et al., 2018; Sickinghe et al., 2019). Whereas HFrEF is related to larger vessels developing after an ischemic event (Sickinghe et al., 2019), HFpEF is gradual, involving the microvasculature of the heart (Lee et al., 2016).

A mechanism contributing to this phenomenon may be through 17β-estradiol (E2). In fact, E2 is able to regulate eNOS activity, increase soluble guanylyl cyclase (sGC) to elevate cyclic GMP (cGMP) concentrations leading to activation of protein kinase G (PKG) (Nevzati et al., 2015). Further, E2 also acts via protein kinases PI3K and Akt signaling (Gourdy et al., 2018). Through p38/MAPK signaling, E2 inhibited the proliferation of VSM cells only in female mice (Pellegrini et al., 2014). Furthermore, the inflammatory regulation of the initial atherosclerotic plaque is prevented by the activation of ER-α, decreasing the deposition of lipoproteins and, consequently, the formation of fatty streaks (Elhage et al., 1997).

With aging, perivascular fibrosis (formation of fibrosis around blood vessels) increases (Nosalski et al., 2020) and this fibrosis formation can be influenced by estrogen (Sickinghe et al., 2019). In women, the inhibition of collagen I and III production occurs through activation of ER-α cardiac fibroblasts by E2, whereas in men the E2 to ER-β binding stimulates collagen production (Dworatzek et al., 2019). Cardiac fibrosis in men occurs due to the androgenic influence of the up-regulation of TGF-β, resulting in extracellular matrix deposition (Kong et al., 2014).

#### Interleukins

Comorbidities such as diabetes, obesity and hypertension share the ability to induce a systemic inflammatory state which was recently shown to be accompanied by a larger deterioration of the cardiac cells function and structure in HFPEF (Mohammed et al., 2012; Paulus and Tschöpe, 2013). In fact, high circulating levels of interleukin-6 (IL-6) and tumor necrosis factor α (TNF-α) were observed in a cross-sectional study of HFPEF patients regardless the sex (Kalogeropoulos et al., 2010).

### Sex Differences in Vascular Aging

Vascular aging increases the risk of CVD, despite being an independent risk factor for age-related diseases. Further, women often present with acute myocardial infarction later than men (Nanna et al., 2019) due the protective role of circulating estrogens against vessel lipid accumulation and endothelial dysfunction (Chakrabarti et al., 2014).

Among the vascular abnormalities presented are decreased vessel elasticity, increased vascular stiffness and pulse wave velocity (PWV), decreased vascular elasticity, increased lumen, increased senescence of vascular cells, compromised vascular homeostasis and, vascular remodeling (Tian and Li, 2014; Donato et al., 2015; Ding et al., 2018; Cao et al., 2020). In addition, endogenous antioxidant capacity is decreased and reactive oxygen species are elevated, altering the redox balance (Gliemann et al., 2016). With aging, large-arterial stiffness and wave reflections increase and this is particularly higher in women (Smulyan et al., 2001; Mitchell et al., 2004).

#### Vascular Cell Senescence

Cellular senescence is heterogeneous and cell specific, triggered by critical stressors including DNA damage, oncogenes, among others (Gliemann et al., 2016). Vascular senescence is a pathophysiological process of structural and functional changes including dysregulation of vascular tone, increased endothelium permeability, arterial stiffness, impairment of angiogenesis and vascular repair (Mitchell et al., 2004).

Vascular endothelial cell (EC) senescence is observed in both inflammation and aging and is associated with vascular dysfunction, leading to CVD in the aging individual (Smulyan et al., 2001). Senescent ECs show attenuated endothelial nitric oxide (NO) production, increased endothelin‐1 (ET-1) production, elevated inflammation, increased expression of adhesion molecules VCAM-1 and ICAM-1 and increased cell apoptosis, as well as increased activation of NF-κB (Colpani and Spinetti, 2019). In fact, a randomized study involving 541 men and women, aged 70–82 years, demonstrated that elevated levels of plasminogen activator and Von Willebrand factor, as markers of EC injury and dysfunction, were associated with lower cerebral blood flow in older adults at high risk for CVD (Mitchell et al., 2004).

Interestingly, a study with cell culture-based bioassay on primary human arterial endothelial cells from both men and women with peripheral artery disease showed that cellular ROS production was higher in women than in men, suggesting increased endothelial oxidative stress (Gardner et al., 2015; Uryga and Bennett, 2016; Jia et al., 2019; Sun and Feinberg, 2021).

#### Vascular Stiffness

The molecular mechanisms involved with vascular stiffness resulting from fibrosis and extracellular matrix are stimulated by vasoactive molecules such as endothelin-1, aldosterone and angiotensin II, commonly associated with aging (Harvey et al., 2016). The pathways related to pathophysiological changes in the vascular endothelium include activation of the transforming growth factor-β1, increased expression and activation of matrix metalloproteinases, galectin-3 overload, SMAD signaling, activation of the renin angiotensin-aldosterone system and, activation of inflammatory and fibrotic signaling pathways (p38 MAPK, TGF-β) (Qi et al., 2011; Yaghooti et al., 2011; Wang et al., 2012; Calvier et al., 2013; Song et al., 2015).

With aging, arterial stiffness increases in both sexes (Mitchell et al., 2004), however, women display a greater stiffening after menopause, which corroborates with the decrease in estrogen levels. Further, with aging, large-artery stiffness and wave reflections increase and this is particularly higher in women (Smulyan et al., 2001; Mitchell et al., 2004). In fact, women taking hormone replacement therapy (HRT) displayed a decrease in the carotid‐femoral PWV (Rajkumar et al., 1997). A study with ovariectomized monkeys showed that 18 months of estrogen treatment was able to reduce arterial stiffness (Adams et al., 1990). Testosterone deficiency in men without CVD is associated with increased carotid-femoral PWV, probably promoting premature vascular aging due to increased arterial stiffness (Vlachopoulos et al., 2014). Another evidence of low testosterone levels is microvascular dysfunction in middle-aged men (Corrigan et al., 2015). Moreover, hypogonadal men who use testosterone replacement therapy show an improvement in arterial stiffness (Yaron et al., 2009).

Wall shear stress is the drag exerted by flowing blood on the vessel wall, playing an important role in the production of vasoactive substances by endothelial cells. Interestingly, a longitudinal observational study reported no significantly differences between sexes regarding mean shear stress in 12 years of observation, however, it was shown that peak shear stress decreases significantly only in men. Further, arterial stiffness increases with aging with women displaying a greater result (+74.5% in women and +28.0% in men) (Irace et al., 2012). In the same way, mean wall shear stress from neck vessels decreased with age and it is significantly higher in females than in males, possibly due to a decrease in flow (Zhao et al., 2015).

In addition, women have less coronary flow reserve than men and have more functional than structural coronary abnormalities (Shaw et al., 2009; Collins et al., 2020). The Working Study Group on micro- and macro- circulation of the Italian Society of Hypertension (SIIA) found that the vascular wall/lumen ratio tends to increase with age in both sexes (Bruno et al., 2018; Rizzoni et al., 2019). Older women have less baroreflex sensitivity than older men, presenting greater carotid artery stiffness (Lacolley et al., 2017). Finally, PWV increases in both sexes, however, it is more pronounced in men over 50 years of age (AlGhatrif et al., 2013). When comparing telomere lengths between men and women, it was found that with advancing age, men with shorter telomeres were more prone to high blood pressure and high PWV values (Laina et al., 2018).

#### Oxidative Stress and Inflammation

Key mechanisms such as oxidative stress and inflammation may contribute to the vascular aging process via decreased testosterone levels in both women and men (Moreau et al., 2020). Augmented ROS, acting as inflammatory mediators, impair endothelial function and worsen arterial stiffness by decreasing NO production, degrading elastin, and increasing collagen and calcium deposition. Even in apparently healthy adults, aging causes a progressive decline in macro and microvascular endothelial function. Furthermore, there are gender differences in the rate of this decline (Moreau et al., 2020).

In fact, male rats treated with testosterone showed increased antioxidant activity (catalase and superoxide dismutase) which was lost with castration (Ahlbom et al., 2001; Kłapcińska et al., 2008; Eleawa et al., 2013). Similarly, in humans, decreased testosterone concentration was correlated with pro-inflammatory cytokines in older men (Barud et al., 2002; Malkin et al., 2004). Moreover, hypogonadal older men who take testosterone supplementation displayed decreased circulating levels of TNF-α and increased levels of IL-10 (Malkin et al., 2004).

In early and late postmenopausal, high testosterone concentrations elevated levels of inflammatory markers and C-reactive protein (Sowers et al., 2005; Maturana et al., 2008; Maggio et al., 2011). Experiments in ovariectomized (OVX) spontaneously hypertensive rats showed an increased relaxation in aorta after treatment with estrogen compared to OVX rats, however, this protection was abolished when the animals received estrogen plus testosterone, activating NADPH-oxidase subunit p47^phox^ and consequently increasing the production of superoxide (Costa et al., 2015).

Moreover, infusion of vitamin C (antioxidant) in women who underwent OVX restored vasodilatory responses to acetylcholine (Virdis et al., 2000) and increased FMD in estrogen-deficient postmenopausal women (Moreau et al., 2013). Aerobic exercise increases FMD in older men, but only postmenopausal women who are receiving E2 therapy change, probably due to oxidative stress (Moreau et al., 2013).

Vascular homeostasis is due to the balance between endothelial vasoconstrictor and vasodilator factors, thus, endothelial dysfunction can result in CVD. Adequate NO synthesis and release by endothelial cells are predictors of a good endothelial response to stimuli (Sandoo et al., 2015). The accumulation of oxidative stressors, common during aging, triggers a decrease in the production of NO, and may be responsible for the inactivation of SIRT1. Consequently, this inhibition interferes with the growth of endothelial cells due to the increase in acetylation of p53 (Ota et al., 2010; Cencioni et al., 2013). Greater suppression of NO contributes to arterial stiffness in rodent models and elderly women, reducing endothelium-dependent dilation (Durrant et al., 2009; Donato et al., 2018).

In advanced aging and in both sexes, oxidative stress and inflammation can modulate endothelial dysfunction by raising blood pressure, blood glucose, obesity and sodium intake. Thus, changes in the endothelium may occur even in the absence of a related disease (Donato et al., 2015; Tesauro et al., 2017). However, the main cause of morbidity and mortality in women is CVD (Collins et al., 2020). Differences in vascular pathophysiology between sexes have been identified in current advanced cardiac imaging techniques (Maas et al., 2011). Common conditions among women such as polycystic ovary syndrome, pre-eclampsia, autoimmune diseases, anemia, hyperuricemia and menopause can accelerate atherosclerosis, promoting endothelial dysfunction (Lian and Keaney, 2010; Collins et al., 2020). In pre-menopause, a decrease in endothelial function begins, accompanied by a worsening after menopause or another period of prolonged estrogen deficiency (Bechlioulis et al., 2010; Moreau et al., 2012). During the menopause transition, due to the loss of the antioxidant, anti-inflammatory, anti-proliferative and dilating effects of estradiol on the vascular wall, there is a decrease in endothelial function and an increase in arterial stiffness (Moreau, 2018).

#### Estrogen

Estrogen influences vascular protection by positively regulating NO (Jespersen et al., 2012; Wanitschek et al., 2016; Collins et al., 2020). Such relationship can be observed in women who have had an ovariectomy, where they develop a reduction in endothelium-dependent dilation (Virdis et al., 2000). In women, estradiol acts on estrogen receptors (ERs) present in the vasculature by releasing NO via eNOS activation through both genomic and non-genomic mechanisms. Moreover, the greater vascular relaxation by estradiol includes greater β-adrenergic and lower α-adrenergic sensitivity (Duckles and Miller, 2010). After menopause, there is an increased risk of CVD due to the loss of this protection and a reduction in the action of the β-adrenergic receptor for vascular tone (Stice et al., 2009).

#### Mineralocorticoid Receptors

Another mechanism contributing to sex differences in vascular aging is through mineralocorticoid receptors (MRs) which act in response to aldosterone by modulating renal sodium reabsorption and then regulating blood pressure (Kim et al., 2018). It was demonstrated that vascular stiffness increases with age in both sexes, however, later in life of females, correlating with the timing of increased vascular MR expression. Further, this augmented vascular stiffness is prevented in smooth muscle cell-specific MR deletion (SMC-MR) deficient males and females. Interestingly, vascular fibrosis increases later in life in females and is attenuated by SMC-MR deletion in males only (DuPont et al., 2021). Angiotensin II (Ang II) signaling modulates constriction in the vasculature and is known to increase with aging (Wang et al., 2010). Of importance, MR contributes to Ang II-induced vasoconstriction only in males whereas in females, the mechanisms are distinct (DuPont et al., 2021).

The renin-angiotensin system (RAS) plays an important role in the development of CVD in aging. Recently, angiotensin converting enzyme 2 (ACE2) was shown to produce angiotensin (Strehler, 1977; Hayflick, 2007; De La Fuente, 2008; López-Otín et al., 2013; Davalli et al., 2016; Bai, 2018; Steverson, 2018) [Ang-(1–7)], vasodilator/antiproliferative peptide witch has opposite effects to Ang II (Kim et al., 2018; DuPont et al., 2021).

Ang II promotes cell senenescence in HUVEC culture, by increasing senescence-associated galactosidase, DNA damage and adhesion molecule expression which were inhibited by activation of Ang-(1–7) through G protein-coupled receptor Mas (MasR) (Wang et al., 2010). Additionally, Ang-(1–7) causes vasodilator effects in rats via activation of angiotensin type 2 receptors (AT(2)R). Interestingly, a study demonstrated that aorta from aged rats displayed increased AT(2)R, MasR, and ACE2, suggesting that Ang (Strehler, 1977; Hayflick, 2007; De La Fuente, 2008; López-Otín et al., 2013; Davalli et al., 2016; Bai, 2018; Steverson, 2018)-mediated depressor effects are preserved in aged animals (Rubio-Ruíz et al., 2014).

Further, resveratrol, an antioxidant drug, was able to protect against arterial aging through reduced activity of the ACE-Ang II axis and stimulation of the ACE2-Ang-(1-7)-ATR2-MasR axis (Bosnyak et al., 2012; Santos et al., 2013; Kim et al., 2018; Romero et al., 2019).

Age-related changes in vascular responses to angiotensin-(1–7) were demonstrated in both male and female mice. In this study, the Ang-(1–7) vasodilatory effect was absent in aorta from old females compared to young females, which was restored by estradiol replacement. Further, the treatment was able to decrease production of ROS and normalize levels of NO. Regarding male mice, Ang-(1–7) induced a dose-dependent vasodilator effect in aorta regardless of whether the artery was from young or old mice (Costa-Fraga et al., 2018).

The endothelin system is also impacted by aging and a role in sex difference has been demonstrated. It is well known that endothelin receptors (ETR) mediate vasoconstriction and vasodilation in arteries (Van Guilder et al., 2007). Interestingly, a study evaluating endothelial function in men showed a key role performed by ET_A_R in vasodilation and vasoconstriction. In fact, older men displayed blunted responses to acetylcholine which was improved by co-infusion of BQ-123 (ET_A_R antagonist), suggesting that, at least in part, reduction in endothelial function with age is through ET_A_R (Westby et al., 2011). In contrast, in postmenopausal women endothelial function decline due to a loss of ET_B_R-mediated dilation, since ET_B_R antagonist (BQ-788) restored vasodilation in postmenopausal women (Wenner et al., 2017).

## Closing Remarks

This review considered many aspects of how biological sex influences cardiovascular pathophysiology. The following conclusions can be made: 1) sex is an important variable that defines cardiovascular structure and function in health and disease; 2) biological sex must be considered for all study designs and approaches; 3) sex steroid hormones define phenotypic responses and therefore, hormonal status should be considered in the design of animal and human experiments, drug protocols and other interventions; 4) when there is a difference between the sexes in cardiovascular pathophysiology, much can be learned by examining the sex that is least or most protected; 5) extrapolation between sexes regarding cardiovascular physiology and pathology should not be made; and 6) drugs for treatment of cardiovascular conditions in elderly men and women should be personalized.
